# A need to integrate pharmacological management for multimorbidity into dementia guidelines in Australia

**DOI:** 10.3389/fpubh.2024.1425195

**Published:** 2024-07-23

**Authors:** Sanket Nagar, Liwei Ma, Yijun Pan, Andrew Liem Hieu Huynh, Edwin C. K. Tan, Liang Jin

**Affiliations:** ^1^The Florey Institute of Neuroscience and Mental Health, Parkville, VIC, Australia; ^2^Florey Department of Neuroscience and Mental Health, The University of Melbourne, Parkville, VIC, Australia; ^3^Applied Research Centre for Disability and Wellbeing, Launceston, TAS, Australia; ^4^Possability Group, Hobart, TAS, Australia; ^5^Department of Aged Care, Austin Health, Heidelberg, VIC, Australia; ^6^Department of Medicine, Austin Health, University of Melbourne, Heidelberg, VIC, Australia; ^7^School of Pharmacy, Faculty of Medicine and Health, University of Sydney, Camperdown, NSW, Australia

**Keywords:** dementia, guideline, multimorbidity, pharmacological management, polypharmacy

## Abstract

**Introduction:**

Pharmacological management is a vital aspect of dementia care. Suboptimal medication prescribing and adverse drug reactions are major causes for ongoing concerns for the quality of care. This review aims to investigate the existence and comprehensiveness of Australian guidelines dedicated to supporting dementia care in the context of pharmacological management.

**Methods:**

Guideline registries and databases (EMBASE and CINAHL) were searched to identify Australian guidelines addressing pharmacological management in dementia care and to uncover barriers and considerations associated with guideline implementation.

**Results:**

Seven Australian guidelines were identified. Barriers to effective implementation were identified at individual, provider, and system levels. None of the identified guidelines provided comprehensive guidance on management of multimorbidity and polypharmacy.

**Discussion:**

Although Australian guidelines are available to guide pharmacological management in dementia, several barriers impede their effective implementation. There is an urgent need for updated guidelines that address the management of multimorbidity and polypharmacy in people living with dementia.

## 1 Introduction

Dementia is a general term for loss of memory, language, problem-solving and other thinking abilities that are severe enough to interfere with daily life (1, 2), including Alzheimer’s dementia (AD, 60–70% of cases), vascular dementia (10–15%), frontotemporal dementia (20%), and Lewy body dementia (5%) (3). In addition, Parkinson’s disease, traumatic brain injury and cardiovascular disease are also considered risk factors for the development of dementia (4). In Australia, dementia is the second leading cause of death and is a public health concern – it increases hospitalizations and placement in residential aged care (5). With an aging and growing population, it is predicted that the number of Australians with dementia will more than double by 2056 to over 1 million people (6). Among these individuals, more than 83% experience profound or severe activity limitation, necessitating support and supervision for communication, self-care, and/or mobility (6).

To overcome the challenges associated with dementia, the use of pharmacological interventions becomes an imperative aspect of care. Although disease-modifying drugs (e.g., aducanumab, lecanemab) have shown promising effects for early-stage AD (7, 8) and have been approved by United States Food and Drug Administration (FDA), they have not been approved in Australia. Current dementia care still focusses on improving and/or maintaining quality of life through the management of specific symptoms (9). Concerningly, an Australian study reported that around 42% of regularly prescribed medications for people living with dementia (PLWD) were considered potentially ‘inappropriate’ (10). For example, antipsychotics and benzodiazepines, are frequently prescribed and administered to manage behavioral and psychological symptoms of dementia (BPSD), despite limited efficacy and significant short- and long-term adverse effects, including death (11, 12). In fact, it is found that only around 10% of psychotropic prescriptions for PLWD in Australia were considered ‘appropriate’ (13). The inappropriate use of psychotropics to manage behavior is often referred to as ‘chemical restraint’. Australia has a largely aging population, with one in three older people with dementia living in supported accommodation (5). The recent findings from the Royal Commission on the aged care sector shed light on the need for substantial reforms, including enhanced training and support for workers, and a strong emphasis on minimizing the use of chemical restraint (14, 15). The issues surrounding the aging population are further heightened in regional and remote areas (16), where resources are already limited.

Another consideration relates to comorbidity and the associated polypharmacy. PLWD have multiple chronic conditions (17), resulting in elevated rates of polypharmacy (18, 19). In Australia, PLWD have an average of five comorbidities (18), with those living in aged care settings prescribed an average of 14.6 medications (20, 21). Often, only one or two medications are specially prescribed for dementia, with most other medications addressing the multimorbidity, e.g., depression, anxiety, cardiovascular disease, incontinence, etc. (19). The high prevalence of multimorbidity and polypharmacy may account for the heightened incidence of adverse drug reactions (ADRs) and poor health outcomes in PLWD. Studies reported that approximately 25–30% of unplanned hospital admissions from aged care settings in Australia can be attributed to ADRs (22), with half of these suggested to be avoidable (23). One study found that around 76% of PLWD in Australia were prescribed at least one medication with a drug–drug interaction (24), with another Australian study reporting 38% of prescriptions had potentially severe interactions, leading to respiratory depression, cardiac arrest, and death (10). Furthermore, due to the protracted and variable progression of dementia, determining the suitability and necessity of medication for comorbidities becomes challenging (25). Medications once considered suitable may become less appropriate as a person’s dementia advances (26). The need for clear and practical guidelines is evident to ensure optimal pharmacological management of multimorbidity and the associated polypharmacy in PLWD.

Together, the issues mentioned above highlight the urgency for Australian-specific guidelines addressing pharmacological treatment in PLWD to manage their symptoms associated with dementia and multimorbidity. Through expert consultation, the National Health and Medical Research Council (NHMRC) has identified priority areas for dementia research and care, including development of interventions that address cognitive impairment and BPSD (27, 28). While these priorities are crucial, pharmacological interventions that address symptoms do currently exist. However, it remains unclear if there is adequate guidance on best practice and proper implementation. Guidelines play a vital role in providing clear and effective directions for treatment. By addressing issues like inappropriate psychotropic use and polypharmacy, potential harm can be minimized, ultimately reducing the strain on healthcare resources, and contributing to policy development and reform. This review aims to identify the current gaps in the guidelines for pharmacological management of PLWD in Australia and synthesize the main barriers to allow for future investigation and research, and to facilitate health practitioners and specialist organizations to take the required steps for guideline formulation.

## 2 Materials and methods

A systematic search was conducted for study selection and data extraction. Relevance was assessed based on titles and abstracts, and pertinent articles were stored in Zotero (version 6.0.30) with duplicates removed. Data extraction and coding was performed in Microsoft Excel (version 16.80) to capture content, focusing on specific barriers.

### 2.1 Search strategy

Although the data for this review is largely qualitative and there are no comparators, the PICO method has been utilized to integrate search terms. PICO has demonstrated effectiveness when adapted for qualitative research and produced greater sensitivity and comprehensiveness of searches (29). Searches were conducted in the EMBASE and CINAHL databases between 01/09/2023 and 08/09/2023 to identify studies published between 2000 and 2023, and only publications in English were considered. Bibliographies of key papers were also reviewed to identify other relevant papers. To identify current Australian dementia practice guidelines, we also searched the Medical Journal of Australia Guidelines, World Health Organization Guidelines, National Institute for Health and Care Excellence (NICE) Guidance, Therapeutic Guidelines, and Guidelines International Network using search term “dementia” (Appendix 1). Targeted web searches were also conducted to reveal any organizational or governmental clinical practice guidelines. There were no restrictions regarding the type of dementia or patient demographic information. No specific quality criterion, such as the impact factor of a journal, was enforced, and the inclusion criteria were not determined by the study design. Instead, papers were evaluated for their relevance, with the nature and quality of evidence being considered as part of the review process.

## 3 Results

**Figure 1:**
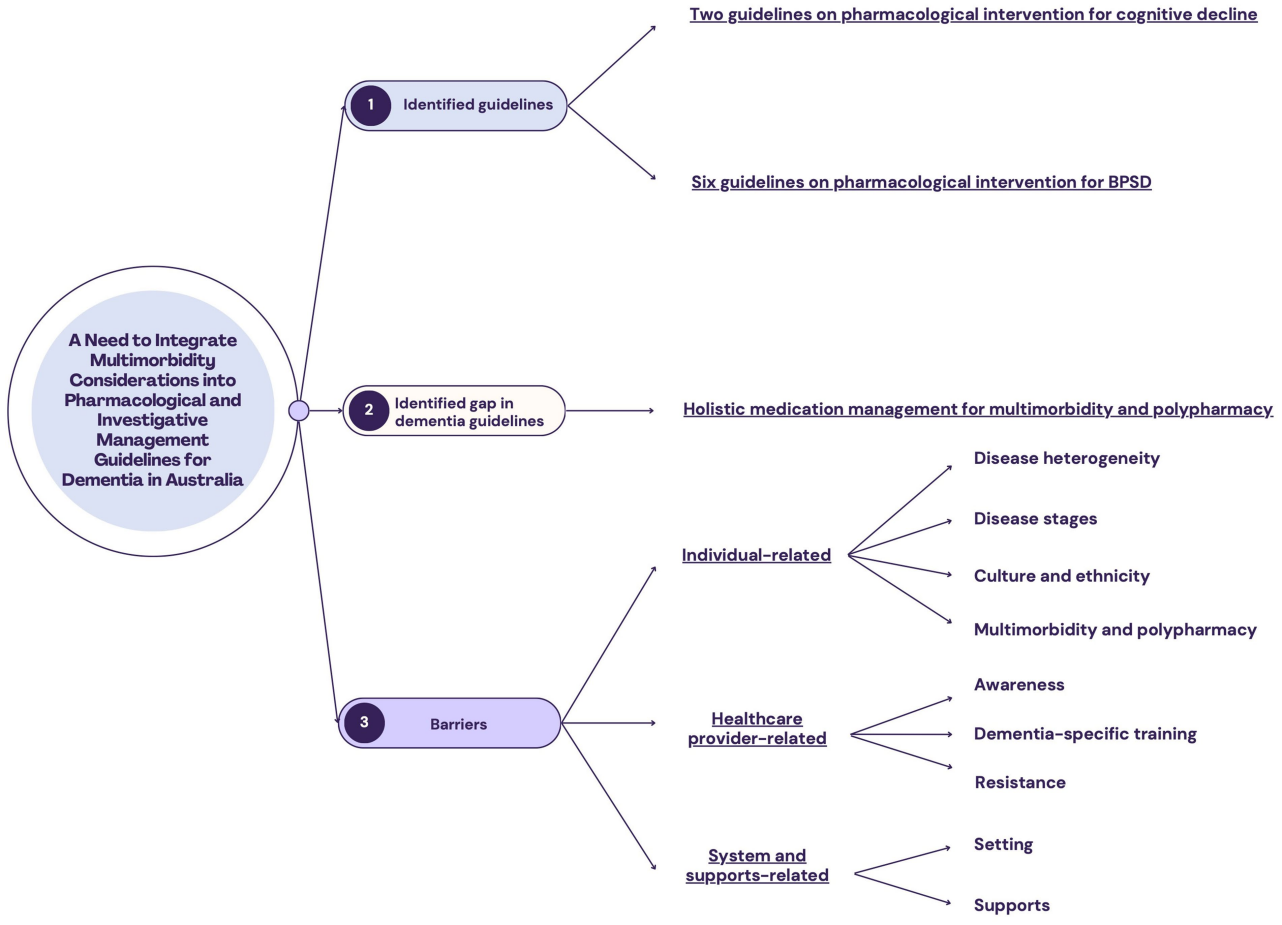
Outline of findings from this literature review. (1) Seven Australian guidelines on dementia care were identified. In the context of pharmacological management, they focused primarily on cognitive decline and behavioral and psychological symptoms of dementia (BPSD). (2) The gap of the identified guidelines in pharmacological management is that comprehensive guidelines on management of multimorbidity and polypharmacy are not available. (3) The barriers to the implementation of dementia guidelines in Australia can be grouped into three perspectives.

The present review identified existing Australian dementia guidelines, highlighted the gaps of these guideline for pharmacological management of multimorbidity, and summarized the main barriers to guideline implementation from three perspectives: (1) individual-related; (2) health provider-related; and (3) system and support-related (outlined in Figure 1).

### 3.1 The identified guidelines

Seven Australian guidelines were identified, which were published between 2012 and 2022 (Table 1). Six were in the form of a report/handbook (30–34, 36), while one was an online resource/webpage (35). Four contain information regarding medication management of the cognitive symptoms of dementia (31, 33–35), with one exclusively elucidating deprescribing these medications (33). Six contain information on the treatment of BPSD (30–32, 34–36). Notably, none of the identified guidelines provide explicit guidance on the management of general medication treatment plans in the context of dementia care, including specific considerations related to multimorbidity and polypharmacy.

**Table 1:** Australian pharmacological guidelines for dementia care.

Publishing Organization	Year	Title	Guideline
Dementia Collaborative Research Centre	2012	Behaviour Management – A Guide to Good Practice: Managing Behavioural and Psychological Symptoms of Dementia (BPSD) (30) *Updated in 2023	BPSD
NHMRC Partnership Centre for Dealing with Cognitive and Related Functional Decline in Older People (Cognitive Decline Partnership Centre)	2016	Clinical Practice Guidelines and Principles of Care for People with Dementia (31)	BPSD; Cognitive symptoms
The Royal Australian & New Zealand College of Psychiatrists	2016	Professional Practice Guideline 10: Antipsychotic Medications as a Treatment of Behavioural and Psychological Symptoms of Dementia (32)	BPSD
The University of Sydney	2018	Evidence-based Clinical Practice Guideline for Deprescribing Cholinesterase Inhibitors and Memantine (33)	Cognitive symptoms
The University of Melbourne	2020	Best-practice Guide to Cognitive Impairment and Dementia Care for Aboriginal and Torres Strait Islander People Attending Primary Care (34)	BPSD; Cognitive symptoms
Therapeutic Guidelines Limited	2021	Dementia (35)	BPSD; Cognitive symptoms
NSW Ministry of Health	2022	Assessment and Management of Behaviours and Psychological Symptoms Associated with Dementia (BPSD): A Handbook for NSW Health Clinicians Providing Services for People Experiencing BPSD (36)	BPSD

Of the guidelines identified, the ‘Clinical Practice Guidelines and Principles of Care for People with Dementia’ (CPG) from the Cognitive Decline Partnership Centre (31) is the gold standard for dementia care in Australia. The CPG was developed by adapting the 2006 NICE guidelines for the United Kingdom’s National Health Service through expert consensus (37) and it was approved by the NHMRC in February 2016 and endorsed by various medical bodies, such as The Australian and New Zealand Society for Geriatric Medicine, and the Royal Australian and New Zealand College of Psychiatrists. Although slated for a formal review in 2021, this has not eventuated. Notably, the 2006 NICE guidelines, which served as the foundation for the CPG, were updated in 2018 (37). This discrepancy in the currency of the guidelines raises concerns about their alignment with current best practices and the latest research developments in dementia care. To address this concern, the update of CPG has recently been funded through the Medical Research Future Fund (MRFF) initiative – Dementia Aging and Aged Care Mission (38), which aims to support older people in maintaining their health and quality of life as they age, living independently for longer, and accessing quality care when they need it. Regardless, although the CPG are considered the Australian gold standard, there are significant gaps when it comes to the pharmacological management of PLWD, which ideally should be addressed in the updated guideline.

The most comprehensive pharmacological guidelines are published by Therapeutic Guidelines Limited (35). Therapeutic Guidelines is endorsed by the Royal Australian College of General Practitioners, the National Prescribing Service, the Royal College of Nursing Australia, and the Society of Hospital Pharmacists of Australia. Therapeutic Guidelines provide detailed information and guidance on the pharmacological management of a wide range of medical conditions, offering a collection of 21 specialty subject drug guides, including over 2,500 clinical topics and 3,500 drug recommendations. The official website emphasizes regular updates, with expert interpretation of evidence and input from a network of medical professionals. Despite this, there is a recurrent reference to seek specialist and expert advice, with a clear condition of use and disclaimer indicating that the information within the guidelines may not be accurate, up-to-date, or exhaustive, and that the guidelines do not constitute professional advice (39). This ambiguity regarding the evidence base makes the practicability and application of these guidelines unclear. In addition, access to these guidelines requires a paid subscription, potentially limiting the extent to which clinicians utilize this resource.

### 3.2 Guideline on pharmacological intervention for cognitive decline

The CPG and Therapeutic Guidelines provide evidence-based recommendations for the use of cholinesterase inhibitors (donepezil, rivastigmine and galantamine), along with the N-methyl-D-aspartate receptor antagonist (memantine) in AD. There is limited clinical recommendation for other forms of dementia, such as Lewy body and vascular dementia.

Regarding clinical guidance for prescribing and monitoring, the CPG offers only general principles. For example, the guidelines note that ongoing treatment is dependent on a “clinically meaningful response…consider[ing] the person’s quality of life, cognitive functioning and behavioural symptoms” (31). However, there is a noticeable absence of specific directives or insights into how medical professionals and carers should interpret clinical aspects like measuring quality of life and cognitive function. Furthermore, there is a lack of clarity regarding the specific indicators to be employed, such as formal neuropsychological testing or assessment of domains of functioning. However, this information is available in the only other NHMRC-approved guideline (33). These guidelines specifically provide clinical information for how to titrate and stop the use of cholinesterase inhibitors and memantine, with clear guidance on titrating regimens, what assessment tools to use to capture clinical change, and what ADRs to monitor during the process. This deprescribing guideline can therefore be used in conjunction with the CPG to ensure best practice, albeit only for discontinuation and deprescribing of medications indicated for cognitive symptoms of dementia.

### 3.3 Guideline on pharmacological intervention for BPSD

Six of the seven identified guidelines specifically address management of BPSD (30–32, 34–36). In Australia, risperidone is the only oral medication that is approved for BPSD associated with AD. Other antipsychotics such as quetiapine and olanzapine are often prescribed off-label (32). The NSW Ministry of Health guidelines provide a hierarchy for medication use, starting with analgesics, then antidepressants, antipsychotics, cholinesterase inhibitors, memantine, benzodiazepines, and anticonvulsants as a last resort (36). The guidelines provide a general overview on each class of medication, including ADRs and recommendations for treatment (36). Similar recommendations are noted in the other guidelines, albeit without a hierarchy of use (31, 32, 34, 35). The CPG offer some suggestions on pharmacological management, noting that medications such as risperidone and olanzapine, which serve as antipsychotics, may be used to address agitation and aggression. Citalopram, a selective serotonin reuptake inhibitor, is recommended for people with dementia who experience agitation, and prior non-pharmacological treatments fail or are inappropriate. The guidelines also mention the necessity of parenteral treatment and highlight that intramuscular administration is preferable to intravenous administration because it is safer (31). It also highlights specific conditions that need to be met before and during treatment, such as consultation with the individual and their family, operationalizing the target behavior, considering comorbidities, and conducting regular reviews every 4–12 weeks (31). Similarly, the guidelines published by Burns and colleagues (30) provide comprehensive detail pertaining to assessment, clinical decision making (including cultural and contextual considerations) and specify treatment for various aspects of BPSD (e.g., Chapters for ‘aggression’, ‘anxiety’, and ‘wandering’). Despite this increased level of clinical guidance in both the CPG and guidelines by Burns and colleagues, there is no specific information on dosages and titration protocols. While this information is available in other guidelines (35), the need to refer to multiple sources may limit applicability and use by medical professionals.

### 3.4 Barriers to guideline implementation

There is a dearth of research specifically addressing appropriate guideline implementation for pharmacological management of PLWD in Australia. Regarding barriers, three overarching themes emerged: barriers that are individual-, healthcare provider- and system/supports-related.

#### 3.4.1 Individual-related

##### 3.4.1.1 Disease heterogeneity

Most research and subsequent guideline recommendations are centralized around AD, which poses challenges when supporting people with mixed or other types of dementia. For example, the CPG do not provide any guidance on the pharmacological management of people with frontotemporal dementia, with no mention of this form within the sections addressing cognitive or behavioral symptoms (31). This is concerning when considering that around 10% of dementia presentation reflects frontotemporal dementia and symptoms often manifest as behavioral changes, which are quite different from AD (31, 40). Even among individuals with the same type of dementia, there can be substantial variability in symptoms and disease manifestation. While guidelines offer valuable information on the severity of dementia and how to manage medication accordingly, the individualized and specialized care required often transcends what guidelines alone can provide (25).

##### 3.4.1.2 Disease stages

One of the central issues highlighted in the literature is the intrinsic complexity of assessing and monitoring the effects of medications on PLWD, who may have varying presentations at different stages of their disease (25, 41). Individuals may respond differently to medications and effects are often subtle and develop insidiously, making it difficult for family, supports, and medical practitioners to assess if the medication is effective (25). This is furthered by that many PLWD, especially within the later disease stages, are unable to express their experiences and self-report medication effects, including ADRs (42). Another aspect to consider is that objective tests to assess the effect of these medications are time consuming to perform. For example, in BPSD, measurements such as the neuropsychiatric index and Cohen-Mansfield agitation index are long and difficult to perform. Quite often for BPSD, these medications are given in a residential aged care setting where you have different staff on who are busy and are unable to give you the time to provide objective responses if there has been a response to medication. Notably, many PLWD discontinue medication due to perceived lack of efficacy and ADRs, further highlighting the individualized factors related to medication tolerance and treatment goals. The importance of a person-centered approach to pharmacological management in dementia care becomes evident, emphasizing the need to tailor medication management to the individual’s treatment goals, considering their unique preferences and values (27, 43).

##### 3.4.1.3 Culture and ethnicity

Three of the identified guidelines explicitly addresses engagement with individuals from different cultural backgrounds (30, 31, 34). Burns et al. considered both culturally and linguistically diverse (CALD) populations and Aboriginal and/or Torres Strait Islander (ATSI) peoples (30). CPG also mentions ATSI in their recommendation 14–18 and CALD in their recommendation 19–21 (31). In addition, Belfrage and colleagues developed a guideline regarding cognitive and dementia care for ATSI people attending primary care (34). More comprehensive CALD/ATSI guidelines are in need as cultural-and ethnicity-related factors have been noted to be associated with dementia prevalence, incidence, presentation, understanding, and service utilization (44). Adding to this, medications may interact differently among CALD groups due to genetic variations. Although there is no clear evidence to confirm this in dementia care, around 25% of new medications approved by FDA have different effects across ethnic groups (45). When considering the lack of diverse representation within clinical trials, this poses concerns about the recommendations provided in guidelines. For example, nearly half of all clinical trials in Australia for dementia actively exclude participants non-fluent in English (46). In line with this, a systematic review and meta-analysis of 99 clinical trials for medications to improve cognitive symptoms of dementia found that only 11% of participants were of non-Anglo/Caucasian background (47).

##### 3.4.1.4 Multimorbidity and polypharmacy

None of the identified guidelines offer specific information on managing multimorbidity, resulting in substantial challenges for clinical decision-making. This issue is compounded by the high likelihood of PLWD having multimorbidity and corresponding polypharmacy. The guidelines fail to address practical aspects of medication management, such as how to monitor regular medications or how to balance the risks and benefits of medication use. Relatedly, a recent systematic review noted nonadherence rates in older adults of up to 38% (48). Factors associated with adherence include age, dementia type, medication prescribed, therapy duration, and presence of other concurrent medication and polypharmacy regimens (49) The latter further emphasizes the need for specific consideration for managing multimorbidity and polypharmacy among this population.

#### 3.4.2 Healthcare provider-related

##### 3.4.2.1 Awareness

The initial challenge in appropriate implementation of pharmacological management guidelines is the potential lack of awareness of guideline availability among medical practitioners. Although there is no Australian-specific data to confirm this, a Canadian study found that only half of the participating medical practitioners were aware of available dementia guidelines (50). Practice trends in Australia also allude to the fact that a lack of guideline awareness may be a contributing factor to poor pharmacological management of PLWD. For example, an Australian study indicated that general practitioners (GPs) do not have a protocol for the assessment of dementia and rather have varying practices across services, and within their own practice (51). While this study only involved 30 GPs and was conducted prior to the release of the CPG, it indicates a lack of uniformity within dementia care. A recent Australian longitudinal study further highlighted the lack of awareness of best practice in dementia care (52), where GPs reflected low awareness of the pharmacological management of BPSD and negative effects of long-term anticholinergic agents. This lack of awareness extends globally, as a 2019 survey of over 14,000 health practitioners indicated that approximately 60% held the erroneous belief that dementia is a normal part of aging (53). While this can be attributed, in part, to the broader issue of insufficient dementia-specific training among health practitioners, this shortcoming could be mitigated by referring to the recommendations presented in guidelines. This suggests that practitioners may not be fully aware of the existence or content of these guidelines.

##### 3.4.2.2 Dementia-specific training

It is important to also acknowledge that dementia-specific training for health practitioners is lacking, especially for GPs, resulting in limited knowledge of contemporary approaches to care and best practice. Health practitioners consistently report a lack of training in dementia (52, 54, 55), including its pharmacological management (47, 56). In the Australian longitudinal study mentioned above, mean self-rated scores on a 10-point Likert scale for knowledge of dementia and confidence in treating the condition were just 5.0 and 5.1, respectively (52). This issue is especially pertinent within the Australian healthcare landscape, where GPs often serve as the first and often only health practitioner, especially in regional and remote settings. In these areas, access to dementia specialists, such as geriatricians, neurologists or psychiatrists, is limited, placing a heavier reliance on GPs for dementia care. Efforts have been made in Australia to proactively engage health practitioners with guideline recommendations, yielding some promising results (57). However, the limited uptake of these recommendations continues to underscore the overarching issue: health practitioners often lack awareness of dementia care guidelines and do not consistently implement them in their clinical practice.

##### 3.4.2.3 Resistance

Another theme identified was medical practitioners’ resistance, which can significantly impede the adoption of new guidelines and practices. This resistance may manifest through both overt and subtle actions, ultimately influencing the uptake of guidelines. Within dementia care, one notable aspect of resistance is therapeutic inertia, characterized by the inclination to continue prescribing medications without a thorough review of their net benefit (25). Furthermore, a cognitive shortcut, the availability heuristic, encourages clinicians to persist with medications based on past experiences, even in situations of uncertainty (25). For example, medical practitioners who supported PLWD reported medications have positive clinical effects at least half of the time, even for persistent and challenging symptoms (54). Among the 26 practitioners interviewed, only two described direct experience with possible severe ADRs from a medication (54). The tendency to prioritize personal clinical experiences and rely on this heuristic could significantly hinder the incorporation of guideline recommendations into practice. Drawing from research looking at the use of chemical restraint on people with intellectual disability, a major barrier to reducing incidence was rooted in resistance from medical practitioners (58). This resistance can be attributed to various factors, such as the perceived need to support the carer by continuing to prescribe medication (25, 58). However, interviews conducted in this study clearly highlighted the overt reluctance among practitioners to decrease ‘inappropriate’ medication prescriptions and to engage in consultations with other allied health professionals (58). This is linked to another important aspect contributing to resistance in medical practitioners, that is the fee-for-service health system structure in Australia rewards practitioners only for face-to-face interactions with patients but not for consultations with other health professionals in primary care. The other health professionals are also not rewarded for engaging into these consultations. This aspect is often dismissed or not realized by health service administrators and researchers.

#### 3.4.3 System and supports-related

##### 3.4.3.1 Setting

Dementia care is multifaceted, and its management can significantly differ based on the specific care setting (59). While in theory, the core principles of medical management should remain consistent, real-world practice often reveals distinct variations. For example, research indicates that BPSD increases during acute stays in hospital (60), suggesting that the care environment itself can influence medical decision-making, such as leading to over-reliance on chemical restraints. Accordingly, the practice guidelines provided by The Royal Australian and New Zealand College of Psychiatrists note that where required due to risk of harm, doctors should follow the ‘common law principle of necessity’ and can engage in a manner that may not be appropriate in the community, including administration of medication without direct consent (32). Moreover, transitions between care settings can introduce further complexity. For instance, when individuals move from their homes to formal care settings, there is often a notable increase in the number of prescribed medications (61), including antipsychotics and benzodiazepines (62). This transition underscores the impact of environment on medication management decisions, highlighting the need for adaptable guidelines that cater to these variations.

In addition, dementia presents specific concerns in rural and remote regions of Australia, where approximately 30% of the nation’s population resides. Rural and remote areas typically have an older demographic, with two out of every five residents affected by dementia (5, 16). Unlike major cities, these regions face escalating challenges related to diagnostic, medical, specialist, and support services (63). The unique environmental, systemic, and societal factors inherent to regional healthcare systems predispose rural and remote communities to suboptimal healthcare access and capacity, resulting in elevated rates of morbidity, mortality, hospital admissions, and extended hospital stays (64). Furthermore, given that GPs frequently serve as the initial and often sole point of contact in these areas, the barriers associated with GPs, as described above, are exacerbated within rural and remote settings.

The CPG and other identified guidelines aim to provide overarching guidance for all major forms of dementia and care across community, residential care, and acute hospital settings. However, it is apparent that the guidelines lack the specificity required to address the unique considerations associated with different dementia types and care settings. Specifically, the nuanced aspects of care are not clear and specific direction is limited. On a global context, there exists guidelines that target medication management and administration within community and residential care settings (65, 66), albeit not specific to PLWD.

##### 3.4.3.2 Supports

Beyond the setting itself, the level of support available to PLWD significantly impacts their care. In acute hospital settings, health practitioners, particularly nurses, are typically highly skilled and appropriately trained to administer and monitor medications. In such environments, specific clinical guidelines might not be as crucial, given the presence of medical consultants who can provide direct guidance. Contrastingly, care settings like residential aged care facilities or home-based care may require more comprehensive support, including clear and accessible guidelines (25, 67). It is well-recognized that the roles and responsibilities of support professionals have expanded considerably, placing them in critical positions of care for PLWD, yet they often lack the necessary skills and capacity to effectively manage the complex and diverse presentations of dementia (25, 67–70). Limited knowledge of best practice is also notable within informal care roles, where more than 50% of informal carers have the responsibility of managing medications for PLWD (71). Concerningly, studies have found that informal carers may change medication without consultation (68) or forget to obtain medications when required (49). Medication-related errors increase with a greater number of medications, often mediated by comorbidities (68), further highlighting the need for guidelines to address this aspect of dementia care.

## 4 Discussion

Multimorbidity and polypharmacy requires urgent attention and updated guidelines, which intersects with other critical areas and exacerbates the identified barriers to optimal care. Despite the existence of seven Australian guidelines, their coverage of medications primarily offers a superficial overview of generic ADRs without providing comprehensive support for clinical decision-making. These guidelines fall short of offering essential guidance on how to balance the positive effects of medication against potential ADRs. Notably, none of the identified guidelines offer specific direction on medication management when multimorbidity is present. While general recommendations advise healthcare providers to remain vigilant about contraindications and polypharmacy, specific guidance on how to adjust dosages of concurrently administered medications and how to effectively monitor and report ADRs is conspicuously absent. This lack of guidance is particularly challenging in the context of dementia, as PLWD may have alterations in pharmacokinetics and pharmacodynamics, rendering them more susceptible to medication-related harm (72). This concern is magnified by the elevated number of medications simultaneously prescribed to PLWD (19, 73). The CPG, for instance, notes that ‘recommendations regarding the use of pharmacological agents…may not apply to those with pre-existing, comorbid [conditions]’ (31). Other guidelines also highlight the importance of considering coexisting conditions and polypharmacy but lack specificity (36).

Typically, clinical practice guidelines for non-dementia conditions rarely address consideration for PLWD, polypharmacy, or the scarcity of evidence regarding the efficacy of treatments for older adults (74). An Australian review of 17 clinical practice guidelines for chronic conditions found that only nine offered generalized considerations for older adults with comorbid conditions (75), while only one addressed the needs of older adults contending with multimorbidity (75). This limited applicability for individuals diagnosed with multiple health conditions, is congruent with international guidelines (76, 77). Additionally, clinical practice guidelines predominantly underrepresent older adults demographically (78). This lack of specific evidence and guidance regarding the management of multimorbidity and polypharmacy may contribute significantly to the underutilization of guidelines by medical practitioners. Furthermore, for vulnerable patients, such as PLWD, reducing polypharmacy is a key priority worldwide (79). Drugs will be discontinued through deprescribing when they are no longer required or to minimize ADRs, thereby minimizing potential harm (80). In the present review, only one guideline focused on the deprescribing of dementia medication (donepezil and memantine), but there is no deprescribing guidelines regarding multimorbidity in PLWD. Therefore, further tools and resources to guide healthcare professionals for deprescribing are required.

An American study with a limited sample of 21 medical practitioners found that disease-specific clinical practice guidelines addressing dementia management could facilitate optimizing practice and was requested by practitioners (25). Some recent international guidelines, such as those for hypertension (81, 82), advocate for an individualized approach for PLWD, although considerable evidence gaps hinder the implementation of these guidelines by clinicians. For example, there is limited knowledge about the effects of antihypertensives in PLWD and how to facilitate shared decision-making when faced with competing health risks. This is concerning, considering that antihypertensives are the second most used medications in PLWD (18). Nevertheless, this understanding can also serve as a catalyst for future guidelines. While it may not be feasible to provide clinical guidance for all potential comorbidities and concurrent medications, priority should be placed on ensuring that the most common comorbidities and medications are addressed in practice guidelines.

This review is not without limitations. Given the expansive scope of the research question and the complexity of the topic, it was not feasible to encompass all aspects of care and barriers comprehensively. There may be specific barriers that have not been addressed, and it is possible that there are broader sociocultural factors influencing medication treatment (e.g., cultural views on aging and older people). Exploring these macro-level barriers falls beyond the scope of this review but presents an avenue for investigation in future research. Despite these limitations, this review was able to highlight urgent gaps in both research and practice, which can be used to inform future guideline development.

We uncovered seven Australian guidelines that address the pharmacological management of PLWD. However, none of these guidelines encompass the holistic management of medication, particularly concerning multimorbidity and polypharmacy, which may impede the practicality of the guidelines. Consequently, there is a clear need for further research assessing the effects of multiple medications among PLWD. Greater use of epidemiological data about PLWD and multiple comorbidities (83, 84) can inform decisions and identify both common and significant conditions that require specific consideration. This information can be used by working groups to subsequently review current guidelines and develop supporting guidelines for dementia care that address multimorbidity and polypharmacy. Such guidelines are valuable tools for health practitioners, equipping them with the knowledge required to make well-informed decisions concerning medication management. By providing guidance on best practice and recommendations on the use of medications, these guidelines can significantly contribute to the mitigation of the associated risks related to medication use and facilitate more optimal outcomes within this population.

## Author contributions

SN: Conceptualization, Data curation, Formal analysis, Investigation, Methodology, Writing – original draft, Writing – review & editing. LM: Conceptualization, Data curation, Formal analysis, Investigation, Methodology, Writing – original draft, Writing – review & editing. YP: Conceptualization, Funding acquisition, Methodology, Project administration, Supervision, Writing – review & editing. AH: Methodology, Writing – review & editing. ET: Methodology, Writing – review & editing. LJ: Conceptualization, Methodology, Project administration, Supervision, Writing – review & editing.

## Appendix 1

PICO question

**Table:**

	Search terms
P	Dementia OR Alzheimer* OR Lewy-Bod* OR Fronto-Temp*
I	Pharmaco* OR medica* AND
C	N/A
O	Guideline OR clinical practice

CINHAL searches

**Table:**

	Query	Limiters/Expanders
S1	Dementia OR Alzheimer* OR Lewy-Bod* OR Fronto-Temp*	Search modes – Boolean/Phrase
S2	pharmaco* OR medica*	Search modes – Boolean/Phrase
S3	Guideline OR clinical practice	Search modes – Boolean/Phrase
S4	S1 AND S2 AND S3	Limiters – Published Date: 20000101-20241231 Narrow by Language: – English Search modes – Boolean/Phrase

EMBASE searches

**Table:**

	Query	Limiters/Expanders
1	(Dementia or Alzheimer* or Lewy-Bod* or Fronto-Temp*).af.	-
2	(pharmaco* or medica*).af.	-
3	(guideline or clinical practice).af.	-
4	1 and 2 and 3	Limit 4 to (English language and yr. = “2000–2023”)

Guideline/Webpage registry searches

**Table:**

	Query	Limiters/Expanders
1	Dementia	n/a
